# Inflammatory protein signatures in individuals with obesity and metabolic syndrome

**DOI:** 10.1038/s41598-023-49643-8

**Published:** 2023-12-13

**Authors:** Fayaz Ahmad Mir, Houari B. Abdesselem, Farhan Cyprian, Ahmad Iskandarani, Asmma Doudin, Tareq A. Samra, Meis Alkasem, Ibrahem Abdalhakam, Shahrad Taheri, Abdul-Badi Abou-Samra

**Affiliations:** 1 Qatar Metabolic Institute, Academic Health System, Hamad Medical Corporation, PO Box 3050, Doha, Qatar; 2 Proteomics Core Facility, Office of the Vice President for Research (OVPR), Hamad Bin Khalifa University (HBKU), Qatar Foundation, Doha, Qatar; 3 College of Health and Life Sciences (CHLS), Hamad Bin Khalifa University (HBKU), Qatar Foundation, Doha, Qatar; 4 College of Medicine, QU Health, Qatar University, PO Box 2713, Doha, Qatar; 5 Laboratory of Immunoregulation, Research Department, Sidra Medicine, Doha, Qatar; 6 National Obesity Treatment Center, Hamad Medical Corporation, Doha, Qatar; 7 Weil Cornell Medicine –Qatar, Doha, Qatar

**Keywords:** Predictive markers, Prognostic markers, Biomarkers, Endocrinology

## Abstract

There is variability in the metabolic health status among individuals presenting with obesity; some may be metabolically healthy, while others may have developed the metabolic syndrome, a cluster including insulin resistance, hypertension, dyslipidemia, and increased risk of cardiovascular disease and type 2 diabetes. The mechanisms contributing to this metabolic heterogeneity are not fully understood. To address this question, plasma samples from 48 individuals with BMI ≥ 35 kg/m^2^ were examined (27 with and 21 without metabolic syndrome). Fasting plasma samples were subjected to Olink proteomics analysis for 184 cardiometabolic and inflammation-enriched proteins. Data analysis showed a clear differentiation between the two groups with distinct plasma protein expression profiles. Twenty-four proteins were differentially expressed (DEPs) between the two groups. Pathways related to immune cell migration, leukocyte chemotaxis, chemokine signaling, mucosal inflammatory response, tissue repair and remodeling were enriched in the group with metabolic syndrome. Functional analysis of DEPs revealed upregulation of 15 immunological pathways. The study identifies some of the pathways that are altered and reflect metabolic health in individuals with obesity. This provides valuable insights into some of the underlying mechanisms and can lead to identification of therapeutic targets to improve metabolic health in individuals with obesity.

## Introduction

The prevalence of obesity (defined as a body mass index [BMI] of ≥ 30 kg/m^2^) has increased significantly posing a serious public health challenge. According to the World Obesity Federation, 813 million adults aged 20 years and older were affected by obesity worldwide in 2020 and it has been estimated that this figure will almost double by 2035^1^ Whilst obesity, defined by BMI, is often associated with multiple health consequences including the metabolic syndrome (characterized by central adiposity, hypertension, dyslipidemia, and hyperglycaemia), type 2 diabetes and cardiovascular disease, many individuals within the obesity BMI threshold do not experience these obesity complications. About one-third of individuals with obesity present with no evidence of the metabolic syndrome and have been referred to as having “obesity only” (OBO). About half of OBO individuals may transition to a state of “obesity with metabolic syndrome” (OBM) over a period of 10 years^2^. Studying the pathophysiological processes underlying OBO and OBM will allow a more in depth understanding that will help better classification of obesity as a disease and potentially identify therapeutic targets for the cardiometabolic consequences of obesity. Previously, we have observed that the OBO and OBM phenotypes have differential miRNA and metabolic signatures^3,4^. In this case–control study, we examined differences in specific proteins, using a high throughput plasma protein-screening assay, between OBO and OBM groups.

## Methods

### Study cohort

**Figure 1:**
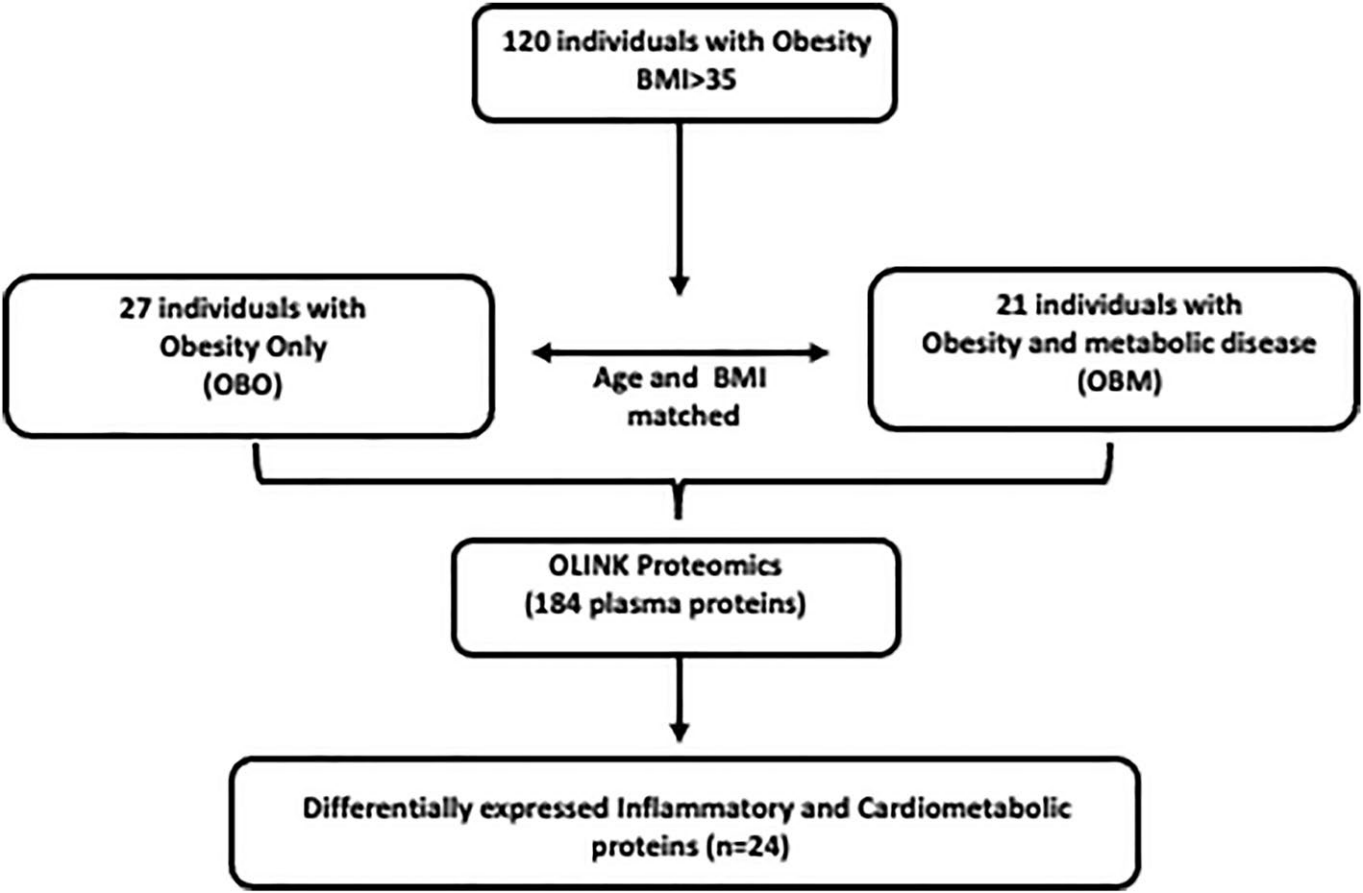
The experimental study design. *BMI* body mass index, *OBO* obesity with no metabolic disease, *OBM* obesity with metabolic diseases.

Participants were recruited at the Qatar Metabolic Institute, Hamad Medical Corporation (HMC), Doha, Qatar. The study protocol was approved by the institutional review board (IRB) of Hamad Medical Corporation (HMC, IRB protocol #16245/16) and all participants provided written informed consent. Throughout the entirety of the study, rigorous adherence to the applicable guidelines and regulations was ensured, thereby guaranteeing that all methodologies employed were meticulously carried out in strict accordance with the institutional guidelines. Obesity was determined according to CDC guidelines. A total of 120, male and female participants aged between 18 to 65 years with BMI ≥ 35 kg/m^2^ were included (Fig. 1). Those with an identified chronic disease or terminal illness were excluded. Participants were classified into two groups: those with obesity only (OBO) and those with obesity and the metabolic syndrome (OBM). The metabolic syndrome was assigned if, in addition to obesity, any 2 of the following co-existed at presentation: triglycerides ≥ 150 mg/dL (1.7 mmol/L), high-density lipoprotein (HDL)-cholesterol < 40 mg/dL (1.03 mmol/L) in men or < 50 mg/dL (1.29 mmol/L) in women, blood pressure ≥ 130/85 mmHg and fasting blood glucose ≥ 110 mg/dL (5.6 mmol/L)^5^. A total of 27 OBO and 21 OBM subjects, matched for age and BMI, were selected for this study (Table 1). Among the 27 OBO subjects, 2 individuals had isolated hypertension, 2 had mildly elevated triglycerides, and 6 had a borderline low HDL-cholesterol.

**Table 1:** Clinical and biochemical traits of the study subjects.

Feature	OBO (n = 27)	OBM (n = 21)	P Value
Age (years)	36.82 ± 5.50	42.76 ± 6.89	0.080
Gender	17(F) 10 (M)	7(F) 14(M)	0.0038
Height (cm)	161.7 ± 7.9	164.9 ± 3.5	0.748
Weight (kg)	115.7 ± 21.5	103.8 ± 33.1	0.161
BMI (kg/m^2^)	41.2 ± 4.9	38.5 ± 2.8	0.228
HbA1c (mmol/mol)	36.1 ± 4.01	58.5 ± 20	0.000
Triglycerides (mmol/L)	1.19 ± 0.45	2.43 ± 1.52	0.001
Total cholesterol (mmol/L)	4.6 ± 1.0	4.3 ± 1.2	0.328
Low-density lipoprotein (LDL)-Cholesterol (mmol/L)	2.7 ± 1.0	2.2 ± 1.1	0.973
High-density lipoprotein (HDL)-cholesterol (mmol/L)	1.4 ± 0.5	1.0 ± 0.2	0.004
Glucose (mmol/L)	5.2 ± 0.6	7.6 ± 3.3	0.004
Creatinine (mmol/L)	65.8 ± 14.1	77.8 ± 33.4	0.131
Insulin (miU/mL)	18.9 ± 14.2	25.7 ± 15.3	0.19
C-reactive protein (CRP) (mg/L)	9.09 ± 5.2	6.7 ± 3.9	0.086
Alanine aminotransferase (ALT) (U/L)	26.8 ± 18.4	31.1 ± 15.9	0.394
Aspartate aminotransferase (AST) (U/L)	20.5 ± 9.3	21.6 ± 6.4	0.632

*OBO* obesity only, *OBM* obesity with metabolic syndrome. Significance was determined by the Student’s t-test.

### Measurements and assays

Height and weight were measured in light clothing without shoes. Fasting blood samples were taken between 7 and 9 AM after at least 12 h of fasting. For serum collection, whole blood was collected via BD Vacutainer Serum Separation Tubes (BD Biosciences, Franklin Lakes, NJ, USA). Blood samples were kept at room temperature for 30–60 min and then centrifuged at 1500 g for 10 min. Following centrifugation, serum was separated and immediately stored at − 80 °C for further use.

Blood biochemistry was performed at the HMC clinical laboratory. Measurements included HbA1c with Turbidimetric Inhibition Immunoassay (TINIA Roche Diagnostics, Mannheim, Germany), glucose by enzymatic reference method with hexokinase (Cobas 6000, Roche Diagnostics International, Switzerland), Total cholesterol, triglycerides, and HDL-cholesterol levels were measured enzymatically using a Synchron LX20 analyzer (Beckman-Coulter, High Wycombe, UK).

### Proteomic assays

Peripheral venous blood (10 mL) was obtained in ethylenediaminetetraacetic acid tubes (Vacutainers; Becton Dickinson) by using a 21-gauge needle to take the sample. Samples were assayed within 30 min of collection at room temperature. Blood samples were then subjected centrifugation (1500 × *g*) for 10 min to separate plasma from whole blood. Following this, plasma was frozen and stored at − 80 °C until subsequent analysis.

Plasma samples were profiled using the Olink proximity extension assays (PEA), 92-plex immunoassay (Uppsala, Sweden) following the standard protocol. Quality control and data normalization were carried out using the Normalized Protein eXpression (NPX) software and every run was checked and validated by the Olink support team in Uppsala, Sweden. Two different protein panels focusing on inflammation and cardiometabolic diseases were used. Protein expression values were calculated as log2 Normalized Protein eXpression (NPX). Olink data that did not pass quality control were excluded from the analyses.

### Bioinformatics

R packages (R Core Team (2023) R: A Language and Environment for Statistical Computing. R Foundation for Statistical Computing, Vienna, Austria. https://www.r-project.org/) for hierarchical clustering (heatmap.2), principal component analysis (PCA), differentially expression analysis (Linear Models for Microarray Data (limma)), volcano plots, WGCNA co-expression networks analyses, gene-ontology biological process (GO) and KEGG pathways enrichment analyses were used through the standalone version of Integrated Differential Expression and Pathway analysis (iDEP) iDEP 0.96 (http://bioinformatics.sdstate.edu/idep96)^6^. Protein–Protein Interaction (PPI) network analysis was conducted using the STRING database v11.5 (https://string-db.org) as described before^7^ and the Human Reference Protein Interactome Mapping Project(HuRI)^8^. PDI analysis was conducted using the Drug-Gene Interaction database (DGIdb, v4.2.0)^9^. To obtain a comprehensive molecular characterization, data were integrated from the two Olink panels for analysis.

### Institutional review board statement

The study was conducted according to the Ministry of Public Health (MOPH) guidelines and approved by the Institutional Review Board Research Ethics Committee of the Hamad Medical Corporation (HMC, IRB protocol #16245/16).

## Results

### Olink analysis revealed differential plasma protein profile in patients with obesity and metabolic syndrome (OBM)

**Figure 2:**
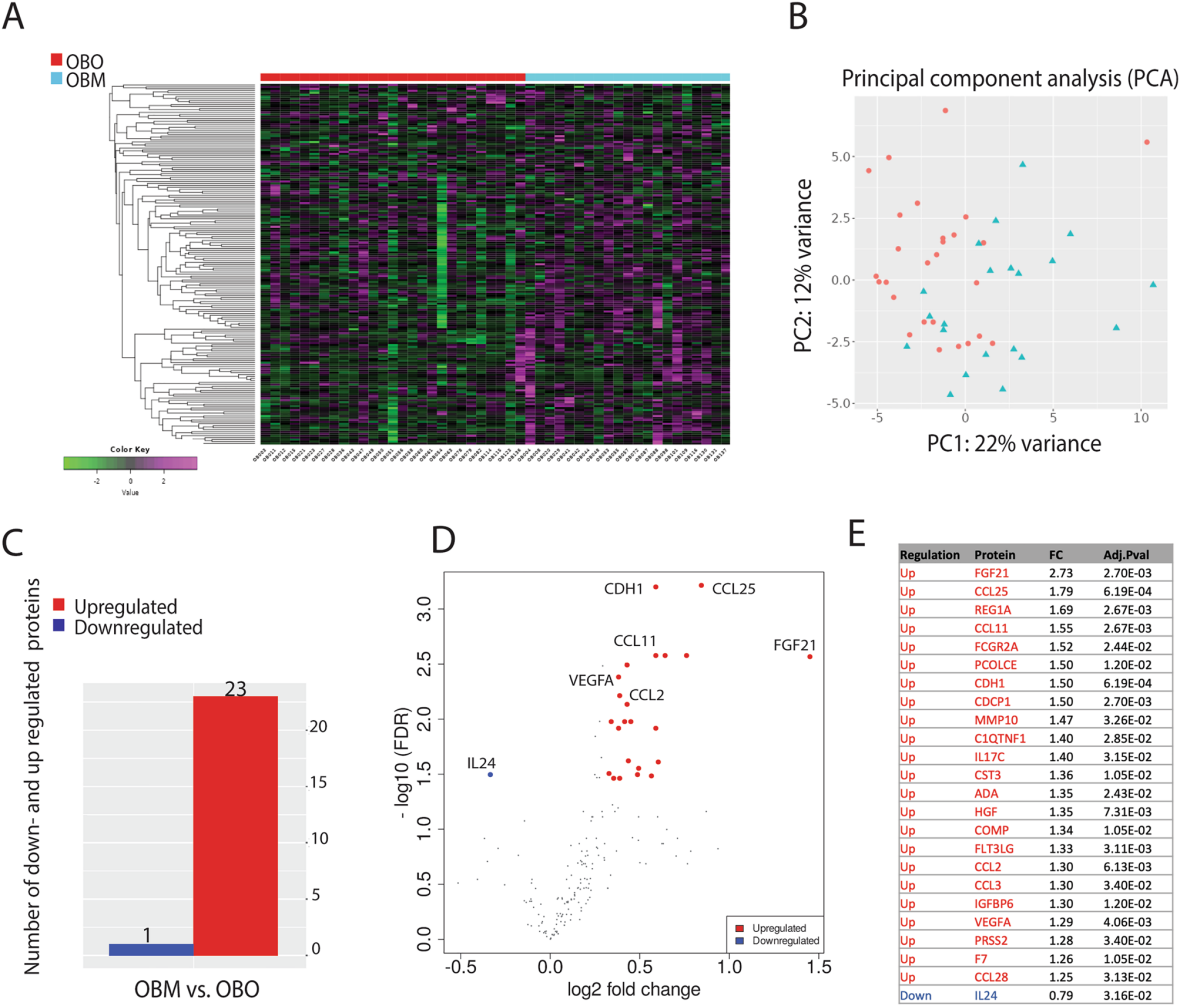
Differential protein expression analysis between OBO and OBM. Differentially expressed proteins (DEPs) were identified from combined Olink inflammation and cardiometabolic panels (184 unique proteins), which was defined as DEPs with more than 1.25-fold change with a P-value < 0.05 and FDR < 0.05. (**A**) Hierarchical clustering based on all 184 proteins assayed using the two Olink panels showed a separation between obese unhealthy patients compared to obese with no metabolic syndrome. The heatmap shows z-scores with cut-off 4, and principal component analysis (PCA) confirmed the separation of the obese cases based on the expression profiles of all proteins (**B**). (**C**) A histogram displaying the number of DEPs across the two groups. The number of the DEPs relevant to all proteins assayed are stated in the panel. (**D**) A volcano plot summarizing DEPs based on log_2_ fold changes across the two groups. Red and blue circles show significantly differential-expressed proteins ≥ 0.6 log_2_ fold change (upregulated) or ≥  − 0.6 log_2_ fold change (downregulated), and p value ≥ 0.05. Differential expression analysis accounted for obesity as the main effect, while correcting for BMI, age, sex, and nationality. (**E**) OBM signature protein list.

Proteomic analysis demonstrated a distinct plasma protein expression profile in individuals with obesity only (OBO) compared to individuals with obesity and metabolic syndrome (OBM). The unsupervised hierarchical clustering revealed that the two panels differentiated between OBO and OBM individuals, as demonstrated by the resulting heatmap (Fig. 2A). Principal component analysis (PCA) from the two groups also disclosed distinct clustering between OBO and OBM samples with first principal component (PC1) representing 22% of the variance. (Fig. 2B). A total of 24 proteins were differentially expressed in OBM subjects compared to OBO subjects (Fig. 2C and D). Only one protein was found to be downregulated, whereas 23 proteins were upregulated at a minimum fold change of 1.25 and a false discovery rate (FDR) cutoff of 0.05. For instance, IL-24 was downregulated in OBM patients, while, FGF21, VEGFA, and others were significantly upregulated in OBM patients (Fig. 2E).

As shown in Fig. 2E, the 23 significantly upregulated proteins in OBM compared to OBO subjects were Fibroblast Growth Factor 21 (FGF21), Hepatocyte Growth Factor (HGF), Insulin-Like Growth Factor Binding Protein 6 (IGFBP6), Vascular Endothelial Growth Factor A (VEGFA), Chemokine (C–C Motif) Ligand 25 (CCL25), Chemokine (C–C Motif) Ligand 11 (CCL11), Chemokine (C–C Motif) Ligand 2 (CCL2), Chemokine (C–C Motif) Ligand 3 (CCL3), Chemokine (C–C Motif) Ligand 28 (CCL28) Fc fragment of IgG, Low Affinity IIa, Receptor (FCGR2A), Procollagen C-endopeptidase Enhancer (PCOLCE), Cadherin-1 (CDHI), CUB domain containing protein 1 (CDCP1), Matrix Metalloproteinase 10 (MMP10), C1q and Tumor Necrosis Factor Related Protein 1 (C1QTNF1), Interleukin 17C (IL17C), Cystatin 3 (CST3), Pancreatic Regenerating Gene 1 (REG1A), Adenosine Deaminase (ADA), Cartilage Oligomeric Matrix Protein (COMP), FMS-Like Tyrosine Kinase 3 Ligand (FLT3LG), Serine Protease 2 (PRSS2), and Coagulation Factor VII (F7) (Supplementary Data 1). To identify the interactions between DEPs and to gain insights into protein network that is dysregulated in OBM, a protein–protein interaction analysis using “String” network tool was performed on the differentially expressed proteins in OBM cases. Visualizing the interactions between DEPs reveals, a network of chemokines (CCL2, CCL3, CCL11, CCL25, and CCL28), interleukins (IL-24, IL-17C) and growth factors (VEGFA, FGF21, HGF) involved in OBM (Supplementary Fig. 1). We further investigated the interactions of our identified DEP with other proteins using Human Reference Interactome (HuRI) where we identified 24 proteins having either a single or multiple protein–protein interractions involving 102 proteins and 276 pathways mainly involving chemokine ligands, growth factors and proteins involved in metabolism, repair and regulating immune responses (Supplementary Figs. 2 and 3). The pathways involving the amino acids phenylalanine, tyrosine, and histidine were found to be implicated in our cohort (Supplementary Fig. 4).

### Functional enrichment analysis and network analysis of DEPs revealed immunological pathways involved in obesity with metabolic syndrome

**Figure 3:**
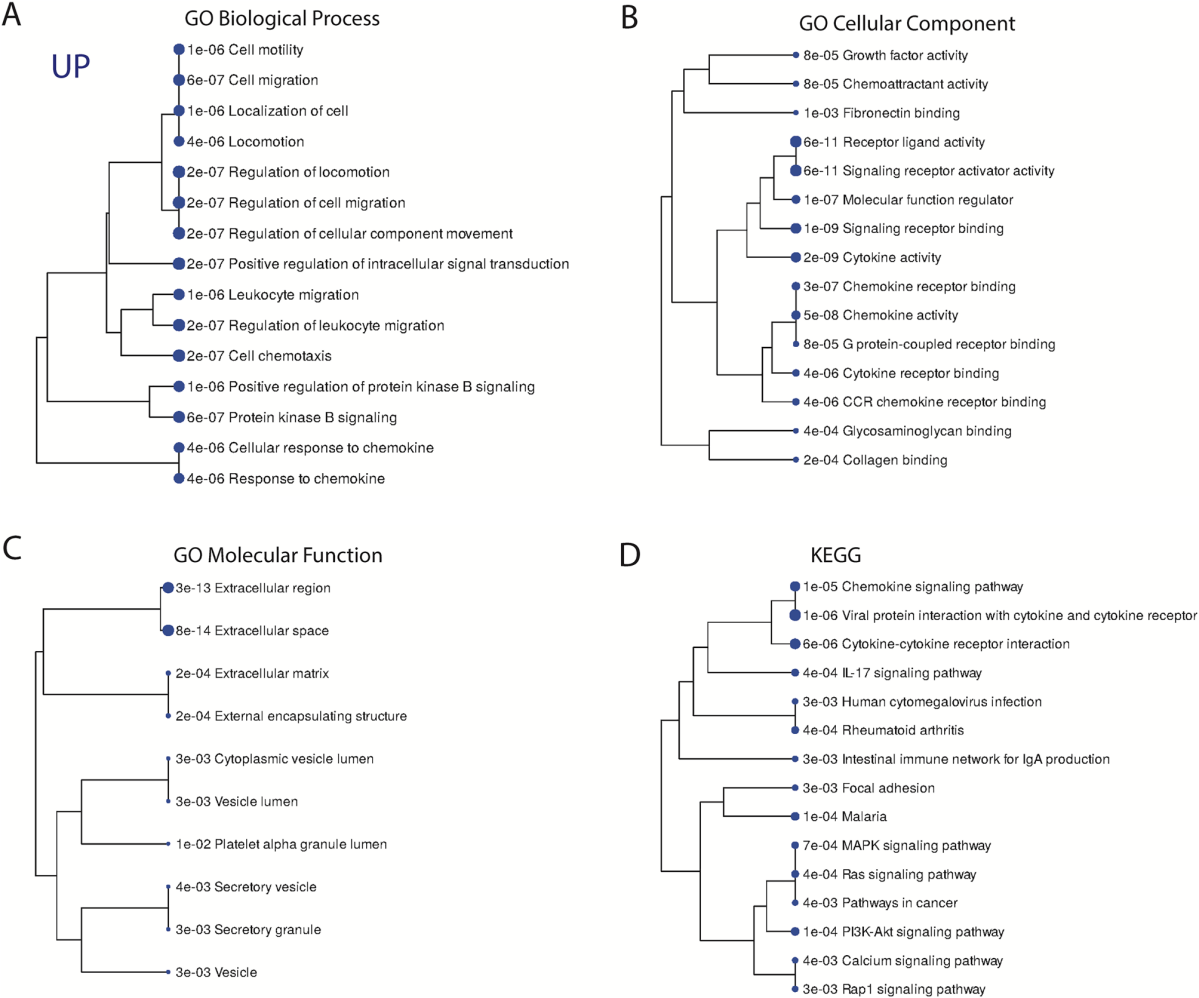
Pathway analysis of DEPs in OBO versus OBM using Gene Ontology (GO), and the Kyoto Encyclopedia of Genes and Genomes (KEGG) databases. (**A**) shows GO Biological processes. (**B**) GO Cellular components. (**C**) GO Molecular Components. (**D**) KEGG pathways. Pathways with a T-test p-value ≤ 0.05 that demonstrate a statistically significant upregulation are represented by blue circles.

Pathways analysis including GO and KEGG^10^ were performed to identify the functional contribution of DEPs in biological processes. DEPs were found to be enriched in pathways related to cellular motility, migration, localization, locomotion, intracellular signal transduction, leukocyte migration, chemotaxis, and protein kinase B signaling. Analysis revealed that 15 immunological pathways were upregulated in OBM cases: chemokine signaling pathway, viral Protein interaction with cytokine and cytokine receptor, cytokine-cytokine receptor infraction, IL-17 signaling pathway, human cytomegalovirus infection, rheumatoid arthritis, intestinal immune network for IgA production, focal adhesion, malaria, MAPK signaling pathway, Ras signaling pathway, pathway in Cancer, PI3k-Akt signaling pathway, calcium signaling pathway and Rap-1 signaling pathway (Fig. 3A–D). These pathways were influenced by growth factors (GF), TNF, IL-1 and MMP-1 (Supplementary Fig. 5).

### Potential pharmacological drugs targeting protein biomarkers of obesity with metabolic syndrome

**Figure 4:**
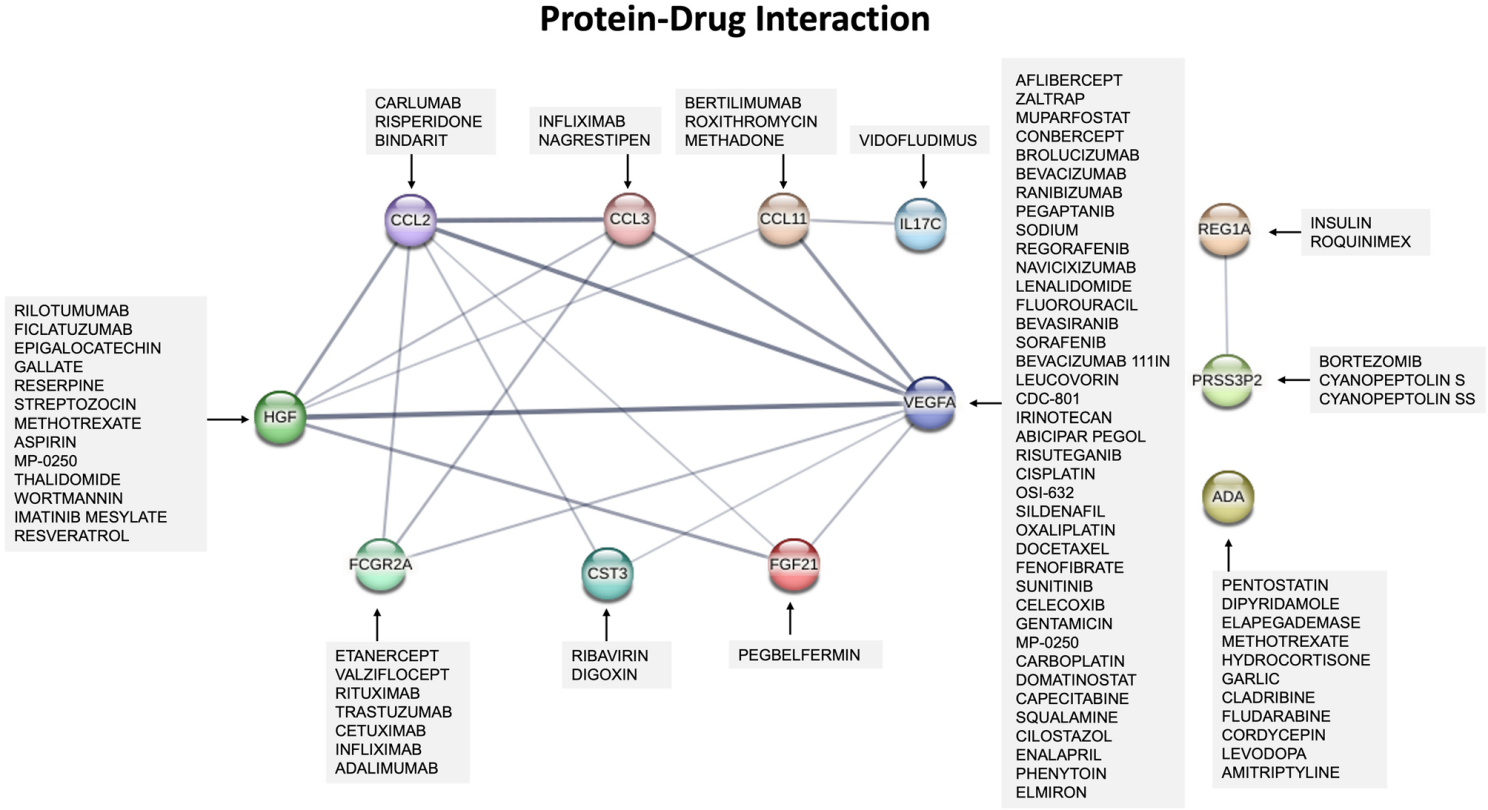
Protein-drug interactions of differentially expressed proteins in unhealthy obese cases. 24 Differentially expressed proteins in OBM were subjected to protein-drug interaction analysis using Drug-Gene Interaction database (DGIdb). Drugs targeting 12 proteins are listed. The 12 proteins with drugs interactions were run in STRING network to assess their protein–protein interactions. The line thickness in the figure indicates the strength of the data support, with a minimum required interaction score of 0.4 for medium confidence.

To gain insights into the role of the identified protein signature, we investigated potential pharmacological drugs that target the protein signatures of OBM cases. An analysis of protein-drug interaction was carried out based on the protein signature using the Drug-Gene Interaction database (DGIdb). We identified 85 drugs that selectively target only 12 proteins, which are among the 24 proteins identified in the OBM protein signature (Fig. 4). Some proteins were found targeted by one drug and others by multiple drugs with a specific interaction type either as inhibitory or antagonistic (Supplementary Data 2).

## Discussion

In our study, we hypothesized and demonstrated differentially expressed proteins and signaling pathways between those with obesity and the metabolic syndrome and those without the metabolic syndrome (Fig. 2). Obesity, usually classified by body mass index (BMI), is characterized by excess body fat accumulation that translates to multiple organ dysfunction and deranged physiological pathways. In particular, many individuals with obesity develop the metabolic syndrome (OBM group) with downstream cardiovascular disease. A significant proportion of individuals, classified with obesity based on BMI, however, do not demonstrate features of the metabolic syndrome (OBO group) and remain metabolically healthy for a prolonged period^11^. The OBO group, still have a higher cardiovascular risk than lean individuals. The OBO status may be a transient status in this group, but demonstrates that there are differential trajectories in cardiometabolic derangements amongst individuals classified with obesity based on current clinical measures. Several studies have identified that the OBM phenotype affects the proteome, gut microbiome, lipidome, metabolome, cytokines, and gene expression^12–19^. These studies provide novel insights into differentiating traits between OBO and OBM individuals with obesity. However, the precise underlying mechanisms that contribute to the differences between metabolically healthy and unhealthy obesity remains poorly understood. Recent work has highlighted the complex pathophysiology of metabolically healthy obesity, and further research is required to better define and understand the metabolic status of obese individuals^20–27^. In this study, we identified several differentially regulated proteins between our OBO and OBM cohort (Fig. 2).

Impaired insulin production coincides with endoplasmic reticulum (ER) and oxidative stress contributing to hyperglycemia in T2DM. Cytokines such as IL-22, IL-23 and IL-24 are upregulated in human T2DM derived islets cultures^28^ and contribute to ER and oxidative stress in pancreatic islet beta cells. The role of these cytokines has been highlighted in murine models of antibody mediated neutralization where improved glucose tolerance was observed^28,29^. The potential protective role of IL-24 remains to be further elucidated in OBO individuals.

It has been suggested that altered expression of proteins involved in tissue remodeling may contribute to the development of metabolic disorders^30–32^. Several of the upregulated differentially expressed proteins (DEPs) identified in our cohort of OBM individuals compared to OBO are chemokine ligands; CCL2, CCL3, CCL11, CCL25, and CCL28 that facilitate innate and adaptive immunity and inflammatory responses (Supplementary Fig. 2). Several studies have shown that elevated levels of CCL2, CCL3 and CCL11 are associated with insulin resistance contributing to the development of T2DM^33^. A recent study has shown that inhibiting the CCL28/CCR10 signaling pathway may be a promising therapeutic approach to improve skin wound healing in a murine model of T2DM induced by obesity^34^. These chemokines can potentially stimulate the recruitment of pro-inflammatory immune cells promoting chronic inflammation, which is associated with impaired glucose metabolism and insulin resistance (Supplementary Fig. 2). Additionally, CCL25 has been linked to hypertension through its effects on the renin–angiotensin–aldosterone system^35^.

Other upregulated DEPs such as Fibroblast Growth Factor 21 (FGF21), Hepatocyte Growth Factor (HGF), Insulin-Like Growth Factor Binding Protein 6 (IGFBP6), and Vascular Endothelial Growth Factor A (VEGFA) are implicated in growth and development signaling pathways targeting NF-κB (Supplementary Fig. 2). In agreement with our results, previous studies have established some associations of FGF21, HGF, and VEGF with metabolic syndrome and its various components. Particularly, FGF21 stimulates the release of leptin and interleukin-6 while suppressing adiponectin^36,37^. Similarly, research has shown that increased HGF levels are associated with higher waist circumference, HDL-cholesterol, elevated liver enzymes, pro-inflammatory gene activation, and insulin resistance^38,39^. Others have demonstrated a positive association between VEGF levels and abdominal obesity, dysglycemia, elevated serum triglycerides, low high-density cholesterol (HDL) as well as elevated blood pressure^40–43^. Additional proteins that are involved in immune response and inflammation include FCGR2A, CDCP1, IL17C, and FLT3LG that commonly interact with VEGF (Supplementary Fig. 2). The upregulation of these chemokines and growth factors in obese with metabolic syndrome group may regulate the growth and differentiation of pancreatic cells and thus play a role in the development of diabetes.

Some of the DEPs reported in this study are involved in pancreatic function and insulin secretion. For example, regenerating islet-derived protein 1 alpha (REG1A) is a protein involved in β-cells regeneration and regulation of pancreatic function, and its dysregulation may contribute to impaired glucose metabolism and an increased risk of diabetes^12–14^. An increase of REG1A in our study points towards inflammatory pancreatic damage. Studies indicate that individuals with T2DM, particularly those with inadequate glycemic control, have an increased levels of cystatin C, clinically used as a marker of renal damage^15^. The elevated cystatin C is indicative of renal impairment in metabolically unhealthy obese due to hypertension, hyperlipidemia and dysglycemia. Similarly, CDH1 also known as e-cadherin regulates endothelial integrity and its increased expression is likely a compensatory mechanism to mediate human β-cell survival^16^. In agreement, PRSS2 a serine protease 2 that is secreted by the pancreas and involved in the digestion of dietary proteins in the small intestine is upregulation in serum following pancreatic inflammation that was elevated in OBM individuals^17^.

Additionally, some of the DEPs are involved in tissue remodeling, repair processes, and collagen biosynthesis and maturation including COMP, PCPE-1, PCOLCE, and MMP10 (Fig. 2). For example, MMP10 is a protease that is involved in extracellular matrix remodeling. It has been suggested that alterations in matrix metalloproteinases (MMPs) and tissue inhibitors of MMPs (TIMPs) contributes to endothelial dysfunction in metabolic syndrome increasing cardiovascular risk and resulting in vascular complications^18^. COMP is a protein that is primarily found in cartilage, tendons, and ligaments and is a large extracellular matrix protein that belongs to the thrombospondin family. Overall, these DEPs are involved in tissue remodeling in the setting of active chronic inflammation in OBM patients interacting to upregulate RANKL and TNF alpha signaling and targeting NF-κB, MAP Kinase and PPAR induced macrophage and neutrophil dependent tissue remodeling (Supplementary Figs. 2 and 4).

Another unifying biological function among reported DEPs is their involvement in glucose and lipid metabolism. C1QTNF1 is a protein that is involved in the regulation of glucose and lipid metabolism, and was suggested as a promising target for early detection, prevention, and treatment of the metabolic syndrome^19^. Correspondingly, ADA is an enzyme that regulates purine metabolism and is responsible for converting adenosine to inosine. Particularly, studies have shown that serum ADA activity is significantly higher in patients with uncontrolled type 2 diabetes and has a correlation with glycemic parameters. Interestingly, serum levels of ADA isoenzymes are significantly elevated in both insulin-dependent diabetes mellitus and non-insulin-dependent diabetes mellitus patients compared to healthy controls^36–38^. Thus, recent evidence suggests that ADA might have a role in insulin signaling and could serve as a marker for insulin resistance in T2DM^39^. The dysregulated glucose and lipid metabolism influence amino acid pathways involving tyrosine, proline and arginine, histidine and phenylalanine that are involved in connective tissue remodeling and immune response (Supplementary Fig. 4)^4^. Finally, Coagulation factor VII is a protein that is involved in the blood coagulation cascade, and its dysregulation may contribute to an increased risk of cardiovascular disease, which is associated with metabolic syndrome. Several studies have highlighted the connection between diabetes and a potential hypercoagulable state, which increases the risk of atherosclerotic complications. These studies have found elevated levels of clotting factors and lower levels of anticoagulants in type 1 and type 2 diabetes, along with platelet hyperactivity^40–43^. Other studies have shown a correlation between elevated levels of coagulation factor VII and various risk factors for cardiovascular disease, dyslipidemia, and insulin resistance^44–47^.

The human protein interactome (HuRI) analysis identified more than 100 different proteins interactions and pathways based on the 24 identified proteins (Supplementary Figs. 2 and 3). The proteins interaction pathways suggest diverse roles in transcription regulation, protein degradation, chemokine pathways, insulin signaling, immune response regulation, healing and repair. In summary, the identified DEPs have diverse biological functions that may influence the susceptibility to and perpetuation of the metabolic syndrome in individuals with obesity including inflammation, tissue remodeling, glucose and lipid metabolism, pancreatic function and insulin secretion. The identified DEPs show interaction potential with over 100 additional proteins that are similary involved in the above mentioned pathways. Noteworthy, several DEPs in our analysis are largely unexplored in human studies in the context of metabolic syndrome in obese individuals.

Our results provide new insights into the underlying mechanisms that contribute to the differences between those with obesity displaying metabolic health and those that manifest the metabolic syndrome. Further research is needed to confirm these findings and to better understand the mechanisms underlying these differences. We highlight the importance of considering not only weight, but also metabolic health in the evaluation and management of obesity, which has implications for the clinical diagnosis, treatment, and prevention among individuals with obesity. Although our explorative study of 184 blood-based inflammatory and metabolic proteins in the blood may add further insight into obesity complications, future studies should assess a larger number of plasma proteins and evaluating the role of the idientified drugs on identified proteins. These drugs may offer advantages in terms of targeted treatment, reduced side effects, and improved patient outcomes compared to drugs targeting other biomarkers. In conclusion, our study sheds new light on the differentiation between obesity with metabolic syndrome and without through the demonstration of a distinctive plasma protein expression profile in obese patients.

### Supplementary Information

Supplementary Figure 1.

Supplementary Figure 2.

Supplementary Figure 3.

Supplementary Figure 4.

Supplementary Figure 5.

Supplementary Legends.

Supplementary Information.

Supplementary Information.

Supplementary Information.

Supplementary Legends.

## Data Availability

The datasets used and/or analysed during the current study will be available from the corresponding author on reasonable request.
