# Supplementary figures and images for: Inflammatory protein signatures in individuals with obesity and metabolic syndrome
Source: Sci Rep. 2023 Dec 13;13:22185. doi: 10.1038/s41598-023-49643-8 (PMC10719383; doi:10.1038/s41598-023-49643-8)

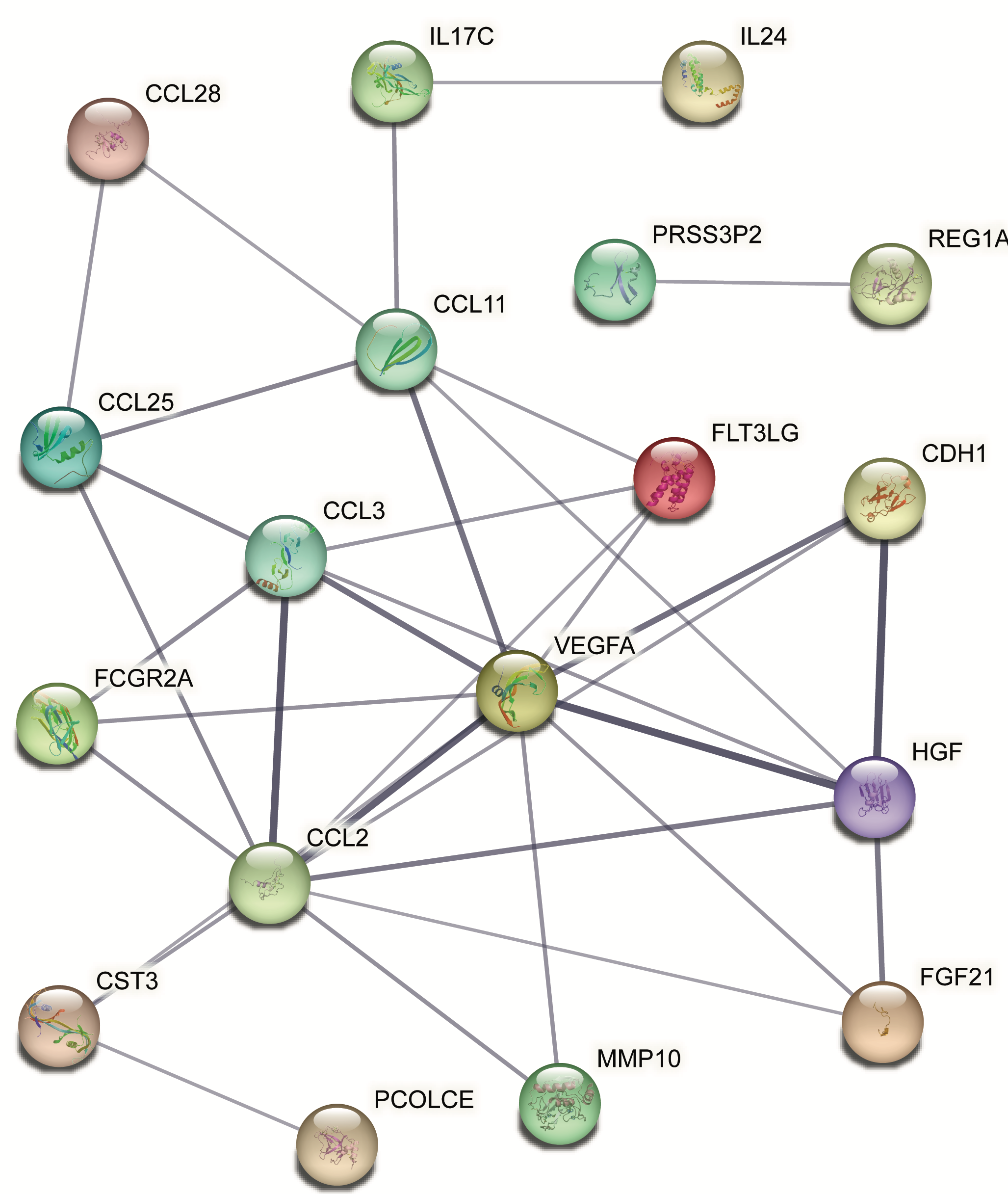

Supplement: Supplementary file 1 — Supplementary Figure 1. [file 41598_2023_49643_MOESM1_ESM.tif]

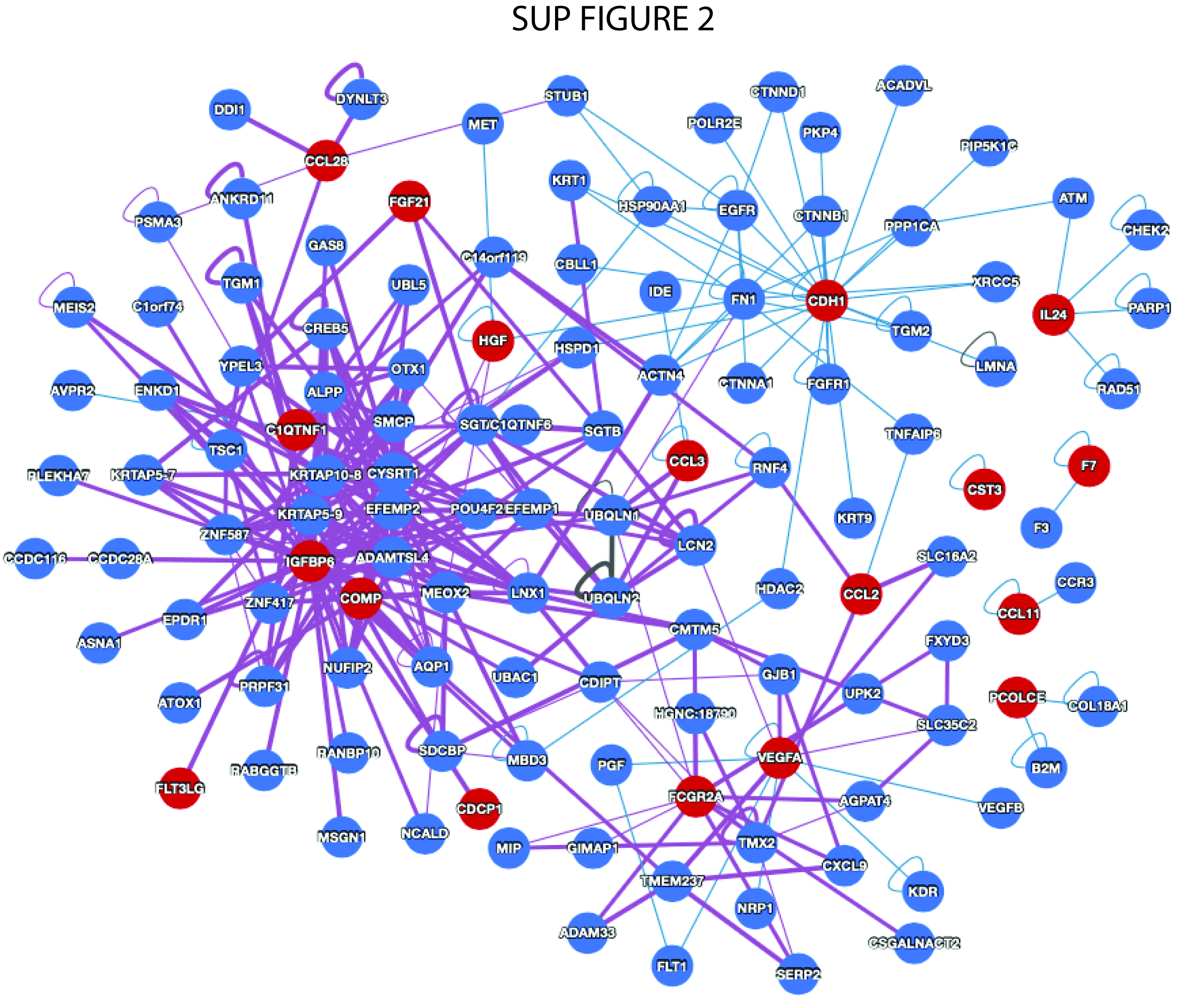

Supplement: Supplementary file 2 — Supplementary Figure 2. [file 41598_2023_49643_MOESM2_ESM.tif]

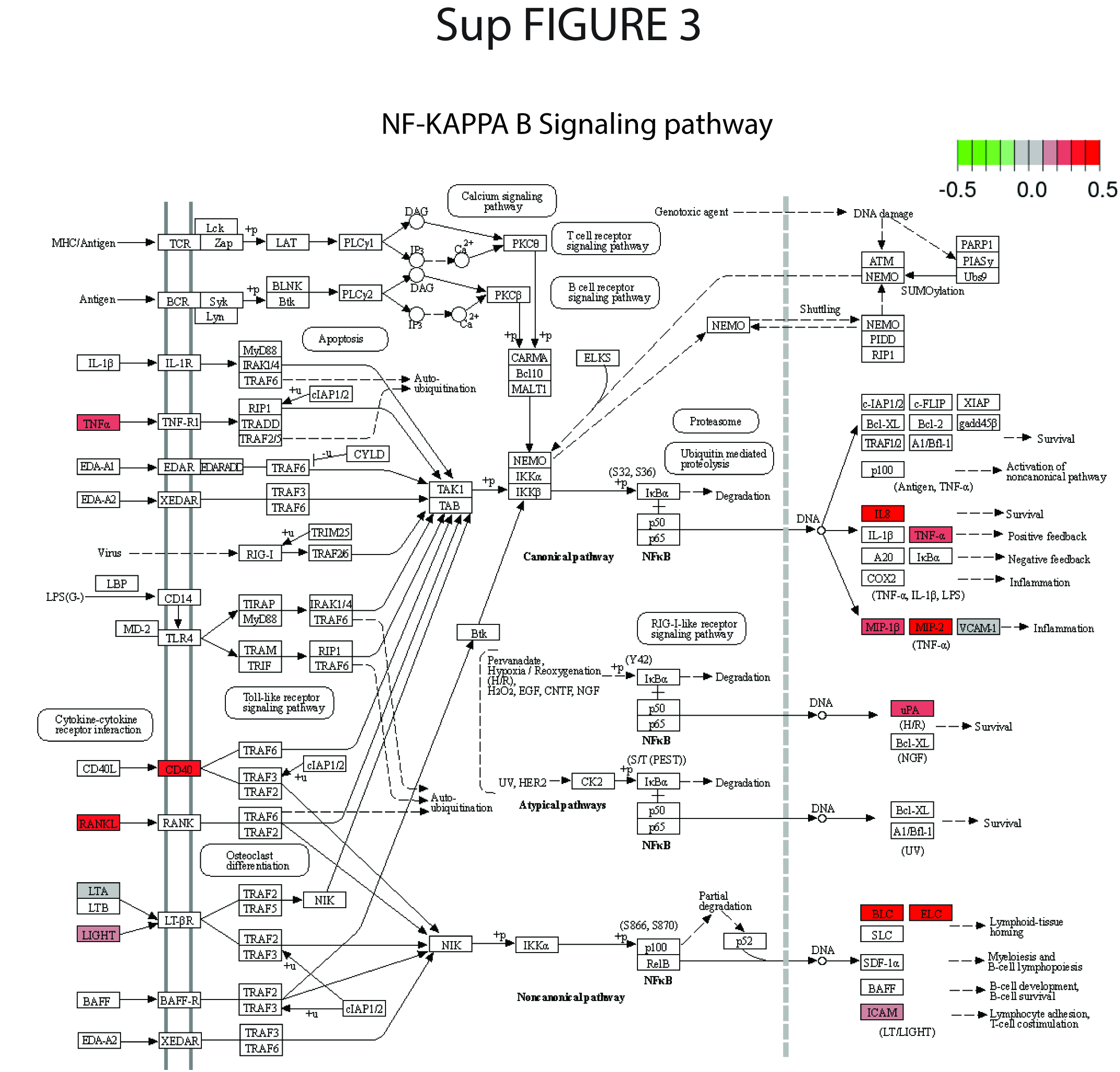

Supplement: Supplementary file 3 — Supplementary Figure 3. [file 41598_2023_49643_MOESM3_ESM.tif]

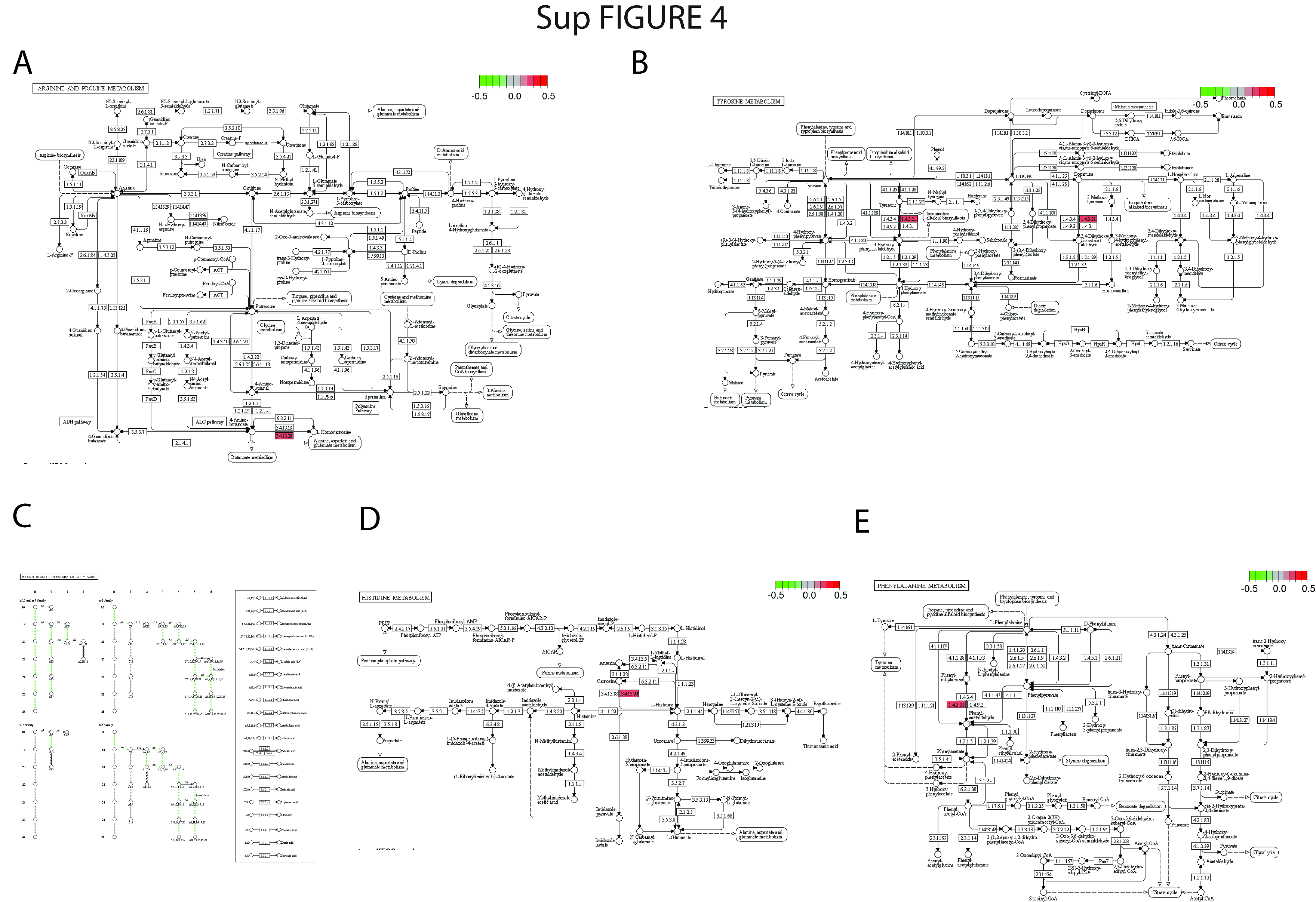

Supplement: Supplementary file 4 — Supplementary Figure 4. [file 41598_2023_49643_MOESM4_ESM.tif]

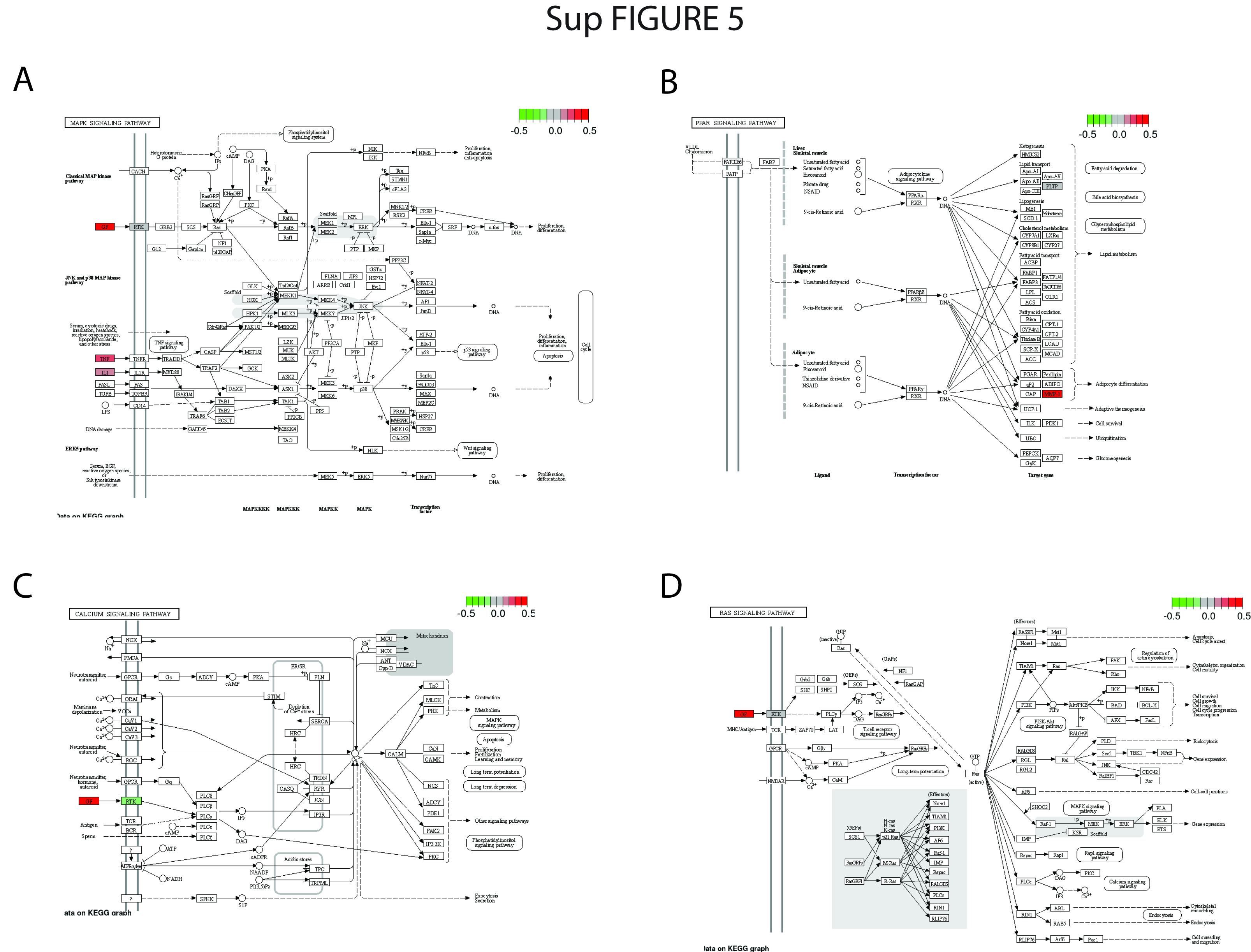

Supplement: Supplementary file 5 — Supplementary Figure 5. [file 41598_2023_49643_MOESM5_ESM.tif]
