# Supplementary material for: Inflammatory protein signatures in individuals with obesity and metabolic syndrome
Source: Sci Rep. 2023 Dec 13;13:22185. doi: 10.1038/s41598-023-49643-8 (PMC10719383; doi:10.1038/s41598-023-49643-8)
Supplement: Supplementary file 6 — Supplementary Legends. [file 41598_2023_49643_MOESM6_ESM.docx]

**Description of Additional Supplementary Files:**

**Supplementary Data 1.** List of differential expressed proteins profile in patients with obesity and metabolic syndrome (OBM).

**Supplementary Data 2.** List of potential pharmacological drugs targeting protein biomarkers of obesity with metabolic syndrome.

**Supplementary Data 3.** List of protein-protein interactions using HuRI network analysis.
