# Supplementary material for: Inflammatory protein signatures in individuals with obesity and metabolic syndrome
Source: Sci Rep. 2023 Dec 13;13:22185. doi: 10.1038/s41598-023-49643-8 (PMC10719383; doi:10.1038/s41598-023-49643-8)
Supplement: Supplementary file 10 — Supplementary Legends. [file 41598_2023_49643_MOESM10_ESM.docx]

**Supplementary Figure 1: Protein-protein interaction using String network analysis.** Identified proteins in OBM were run in string network to assess their interactions. Line thickness indicate the strength of data support. Minimum required interaction score was at 0.4 medium confidence.

**Supplementary Figure 2: Protein-protein interaction using HuRI network analysis.** Identified proteins in OBM were run in HuRI network to assess their interactions. Line thickness indicate the strength of data support.

**Supplementary Figure 3:** NF-KAPPA B Signaling pathway activation in obese unhealthy patients.

**Supplementary Figure 4: Signaling pathways altered in OBM.** **A.** Arginine and Proline metabolism. **B.** Tyrosine metabolism. **C.** Biosynthesis of unsaturated fatty acids. **D.** Histidine metabolism. **E.** Phenylalanine metabolism.

**Supplementary Figure 5: Signaling pathways altered in OBM.** **A.** MAPK signaling pathway. **B.** PARP Signaling pathway. **C.** Calcium Signaling pathway. **D.** RAS Signaling pathway activation in obese unhealthy patients.
